# Supplementary material for: A UMLS-based spell checker for natural language processing in vaccine safety
Source: BMC Med Inform Decis Mak. 2007 Feb 12;7:3. doi: 10.1186/1472-6947-7-3 (PMC1805499; doi:10.1186/1472-6947-7-3)
Supplement: Additional file 13 — RAP application directory. Sets up directory for RAP (RDF application for PHP) source code [file 1472-6947-7-3-S13.gz › rap/doc/phpdoc/__filesource/fsource_dataset__datasetNamedGraphMem.php.html]

xml version="1.0" encoding="iso-8859-1"?


File Source for NamedGraphMem.php


# Source for file NamedGraphMem.php

Documentation is available at NamedGraphMem.php

```
1. <?PHP
2. // ----------------------------------------------------------------------------------
3. // Class: NamedGraphMem
4. // ----------------------------------------------------------------------------------


8. /**
9. * NamedGraph implementation that extends a {@link MemModel}
10. * with a name.
11. *
12. * <BR><BR>History:<UL>
13. * <LI>05-02-2005                : First version of this class.</LI>
14. *
15. * @version  V0.9
16. * @author Daniel Westphal <http://www.d-westphal.de>
17. *
18. * @package     dataset
19. * @access    public
20. ***/
21. class NamedGraphMem extends MemModel
22. {

24. /**
25. * Name of the NamedGraphMem
26. *
27. * @var         string
28. * @access    private
29. */
30. var $graphName;


33. /**
34. * Constructor
35. * You have to supply a graph name. You can supply a URI
36. *
37. * @param  string
38. * @param  string
39. * @access    public
40. */
41. function NamedGraphMem($graphName,$baseURI = null)
42. {
43. $this->setBaseURI($baseURI);
44. $this->indexed = INDEX_TYPE;
45. $this->setGraphName($graphName);
46. }

48. /**
49. * Sets the graph name.
50. *
51. * @param  string
52. * @access    public
53. */
54. function setGraphName($graphName)
55. {
56. $this->graphName=$graphName;
57. }

59. /**
60. * Return the graph name.
61. *
62. * @return string
63. * @access    public
64. */
65. function getGraphName()
66. {
67. return $this->graphName;
68. }
69. }
70. ?>
```

Documentation generated on Thu, 7 Jul 2005 13:42:16 +0200 by phpDocumentor 1.3.0RC3
